# Function2Gene: A gene selection tool to increase the power of genetic association studies by utilizing public databases and expert knowledge

**DOI:** 10.1186/1471-2105-9-311

**Published:** 2008-07-17

**Authors:** Don L Armstrong, Chaim O Jacob, Raphael Zidovetzki

**Affiliations:** 1 Department of Cell Biology and Neuroscience, University of California, Riverside, CA 92521, USA; 2 Department of Medicine, University of Southern California School of Medicine, Los Angeles, CA 90033, USA

## Abstract

**Background:**

Many common disorders have multiple genetic components which convey increased susceptibility. SNPs have been used to identify genetic components which are associated with a disease. Unfortunately, many studies using these methods suffer from low reproducibility due to lack of power.

**Results:**

We present a set of programs which implement a novel method for searching for disease-associated genes using prior information to select and order genes from publicly available databases by their prior likelihood of association with the disease. These programs were used in a published study of childhood-onset SLE which yielded novel associations with modest sample size.

**Conclusion:**

Using prior information to decrease the size of the problem space to an amount commensurate with available samples and resources while maintaining appropriate power enables researchers to increase their likelihood of discovering reproducible associations.

## Background

Many common disorders have genetic components which convey increased susceptibility. While linkage and association analyses have been quite successful in identifying rare variants with high penetrance, such as in Huntington's disease [1] and some forms of cancer [2], the ability of these approaches to detect common variants with more modest effects (less penetrance) is limited. Frequent alleles with modest genetic effects play important roles in the susceptibility to most diseases. For example, most autoimmune disorders involve multiple alleles of different genes with individual low penetrance which also interact with environmental factors to produce the final disease phenotype. Dissecting the disease phenotype into the individual genes and associated alleles that are responsible is crucial for understanding the mechanism of the disease and ultimately developing treatment modalities [3].

To this end, many researchers have been using genome-wide approaches to locate Single Nucleotide Polymorphisms (SNPs) that are in disequilibrium with a disease-causing allele, or associated with a disease phenotype [3]. Unfortunately, the requirement to completely scan the entire genome with sufficient SNP coverage required to achieve an appropriate study-wide power as well as the concomitant increase in number of subjects required to overcome the multiple-testing effect makes this type of study prohibitively expensive. Indeed, many association studies are under-powered, which leads to low reproducibility, compounded additionally by publication bias [4-7].

There are two general methods to reduce the number of SNPs tested in a search for variants which convey susceptibility. The first is to reduce the number of SNPs necessary to cover the entire genome by maximizing the information conveyed by each individual SNP. This process involves the elimination of redundant SNPs whose state is already determined with high probability by other SNPs in the study [8,9]. The second method is to use prior available information to select genes and genomic regions likely to be involved in a disease and testing the most likely genes and regions in preference to the least likely. While this approach does have advantages over a whole-genome study, specifically in financial cost, time required and a smaller number of subjects needed to assure reasonable power, it does have disadvantages in that it does not select candidate genes which are not associated with biological functions or genomic regions currently thought to be related or linked to a disease.

In order to determine which genes or genomic regions are likely to be associated with the disease, it is necessary to connect genes and genomic areas with available literature on the disease and disease-associated pathways. The implementation we present here relies on experts to build a list of keywords and genomic areas from the available literature and expert knowledge coupled with publicly available databases to connect keywords and genomic areas to specific genes. An alternative using automated information extraction techniques which do not rely on expert knowledge is presented in the discussion. This experimental design, where the genes (and consequently, SNPs within those genes) are selected using prior available information allows for the determination of the prior probability that a particular gene is associated with a disease.

Once specific genes have been selected, the SNPs used in the study need to be selected. A popular class of methods is the tagSNP methods, which attempt to reduce the number of SNPs while maximizing the information that each SNP represents by grouping SNPs that are in linkage disequilibrium with each other and in the same phase (see [8,9] for a comparison of different methods). The genes suggested by our program and its associated method do not necessarily require such powerful techniques, but discarding redundant SNPs will be useful in maximizing power while reducing cost. Beyond discarding non-informative SNPs, special importance should be placed on functional SNPs as they are more likely to actually represent the disease allele in question.

The method presented here uses a combination of automated and manual approaches to maximize the power of a study using SNPs. The method has the following steps:

1. Use expert knowledge and literature to identify a set of keywords (biological functions and genetic regions) which have high prior probability of being associated with the disease.

2. Use publicly available databases to select genes that reference the set of keywords.

3. Rank the identified genes based on their prior probability of association using the selection results and expert knowledge.

4. Choose an appropriate number of genes for SNP selection and association studies based on the number of cases available, monetary, and time constraints of the study while maintaining acceptable study-wide power.

5. Conduct study.

6. Analyze results, optionally using the prior probability of association obtained during genetic selection.

## Implementation

**Figure 1:**
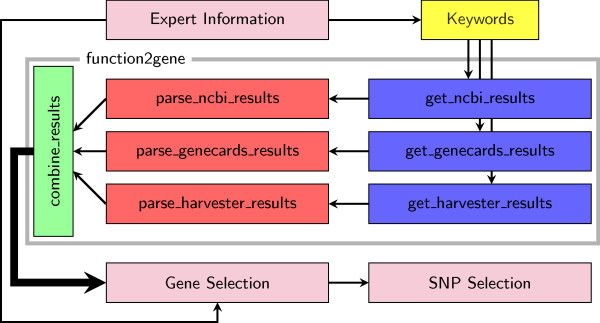
**Schematic of the gene selection process**. Flow chart of the separate methodology and the scripts which enact the methodology, as described in the introduction and implementation sections.

The application is separated into a series of separate scripts, each of which has a specific function, and operate in a specific order (see Figure 1).

Once a set of keywords (biological functions and genomic locations judged to be associated or otherwise involved with the disease) has been identified by expert knowledge of the disease in question, publicly available databases (currently NCBI, GeneCards, and Harvester are defaults, but Uniprot and Ensembl are also supported) are queried in series with delays between each query as appropriate for each database to avoid overloading them. Because there is no well documented common interchange format for these databases, the get_series of scripts download the HTML (or XML) which the search requests generate and save it. To avoid putting unreasonable demands on the databases, the get_scripts limits the number of requests they make per minute.

After all of the HTML (or XML) has been retrieved, the next series of scripts (parse_) are run which use the HTML::Parse (or XML::Parser::Expat) module to extract the gene name, accession number for the reference sequence, genomic location, alias(es), function(s), and description.

These files are then combined into a single file by combine_results that tracks what database and keyword resulted in which genes. The aliases from all databases are joined. Gene descriptions are retrieved from each database; the longest description is retained in the final list. For example, Harvester, when queried with "adhesion", returns VCAM1 (amongst others). Harvester also returns VCAM1 when queried with "inflammation". GeneCards also returns VCAM1 for these two queries. combine_results would then indicate that GeneCards returned VCAM1 twice, Harvester returned VCAM1 twice, and it was returned for "adhesion" and "inflammation". At the same time three separate weighting procedures are applied to order the genes. First, a simple approach, dubbed "rzscore", simply counts the number of times that a gene was returned by unique search terms, and orders the results from most number of term matches to least. The second, allows user-specified weights to be applied to each keyword, and orders the results by the sum of the weights of the corresponding keywords which returned a result. The third method automatically weights the keywords to avoid over-counting keywords which are entirely subsets of other keywords. It does this by dividing the number of results returned by a keyword by the sum of the number of results returned by that keyword and any other keyword, including itself.

To facilitate easier use of this collection of meta-search utilities, a script which binds them all together has also been provided, called function2gene, which takes a set of keywords, an optional set of databases to query, and returns the complete tabulated results to the specified directory.

By using perl and existing modules (WWW::Mechanize, HTML::TreeBuilder and XML::Parser) the actual number of lines (and resulting implementational complexity) of the codebase is greatly reduced, as custom code to deal with form submission as well as HTML and XML parsing did not need to be written. This approach also allows the scripts to be slightly less dependent on the exact output format of the sites which are searched. Splitting the retrieval and parse stages also allows for extracting additional information from the search results by modifying the parser and reparsing the results without waiting to retrieve results from the remote databases again.

## Results and discussion

The system described above was utilized to generate a list of genes which were then used to select SNPs in a study of childhood-onset Systemic Lupus Erythematosus (SLE). SLE is a debilitating multi-system autoimmune disorder affecting ≈ 0.1% of the North American population. An initial search using a set of 31 keywords (consisting of biological functions and chromosomal regions) selected by expert knowledge returned 6798 genes with various contributions from the three databases used (Table 1). It is important to note that the results obtained are temporally-sensitive; as databases are updated different sets of genes will be returned. In every case a single database did not retrieve all the genes found by other databases, demonstrating the need to query multiple databases. The substantial contribution made by each database in identifying the candidate genes demonstrates that each of the databases is required to maximize the number of candidate genes discovered, though there are likely results which are still not captured by the set of databases queried. As new databases come into prominence, Function2Gene can be extended to query them as well. The top 1204 genes (of which 836 were returned by GeneCards, 699 by Harvester, and 135 by NCBI) were used to select 9412 SNPs. The number of genes to select was dictated by the capacity of the chip (≈ 10,000 SNPs), and a decision to have approximately ten SNPs per gene on average. The choice of SNPs to genotype within the selected genes was based on available information from databases including the Human Haplotype Mapping Project (HapMap) with priority given to SNPs with high heterozygosity in two or more relevant ethnicities and to SNPs representing amino acid coding variants. The selected SNPs were then cross-checked against the accumulated SNP validation test results available at our industrial collaborator (ParAllele Biosciences), an active participant of the International HapMap project.

**Table 1:** Results of gene selection process

Keyword	GeneCards	Harvester	NCBI	Total Unique
12q24	355 (90)	217 (6)	276 (25)	391 (337)
16q12	108 (50)	34 (1)	65 (9)	118 (101)
1q23	251 (125)	94 (11)	135 (29)	295 (224)
1q25	144 (65)	50 (3)	94 (20)	167 (117)
1q31	96 (42)	32 (3)	58 (7)	106 (60)
1q41	69 (28)	23 (2)	54 (13)	85 (42)
1q42	212 (66)	107 (6)	153 (22)	241 (190)
2q35	107 (40)	39 (2)	75 (9)	120 (62)
2q36	92 (30)	50 (4)	53 (6)	104 (47)
2q37	217 (62)	97 (4)	156 (12)	233 (197)
4p15	100 (40)	40 (1)	68 (8)	110 (77)
4p16	219 (72)	114 (11)	165 (23)	257 (215)
6p11	19 (12)	6 (2)	9 (1)	23 (15)
6p12	114 (48)	44 (3)	82 (18)	136 (82)
6p21	749 (162)	321 (23)	597 (31)	809 (584)
adhesion	918 (428)	711 (221)	-	1139 (575)
antigen	712 (298)	787 (373)	-	1085 (0)
apoptosis	1013 (555)	682 (224)	-	1237 (564)
chemokine	168 (95)	118 (45)	-	213 (31)
chemotaxis	148 (62)	132 (46)	-	194 (23)
coagulation	145 (73)	111 (39)	-	184 (26)
complement	253 (155)	151 (53)	-	404 (119)
cytokine	531 (256)	439 (164)	-	695 (222)
immunity	132 (101)	125 (94)	-	226 (26)
immunodeficiency	562 (485)	103 (26)	-	588 (213)
inflammation	159 (103)	119 (63)	-	222 (34)
phagocytosis	64 (36)	44 (16)	-	80 (11)

Total Unique	5466 (1925)	3937 (1106)	2025 (204)	6798 (2403)

Number of genes returned for a subset of the keywords used in the example study [10] for each database (GeneCards, Harvester and NCBI). The genes returned by a specific keyword and only this database are in parenthesis. The Total Unique column gives the total number of unique genes for each keyword with unique genes returned only by this keyword in parenthesis. The Total Unique row gives the total number of unique genes for each database with genes returned only by this database in parenthesis. The intersection of the Total Unique row and the Total Unique column gives total number of unique genes with genes returned by only one database, and the number in parenthesis gives the number of genes returned by only one database and only one keyword. - indicates that a keyword was not used with a database. As some composite keywords used in the study [10] were omitted for clarity, the totals in this Table reflect the total number of genes retrieved, not the sums of the columns.

Using the selected SNPs, 251 nuclear families consisting of both parents and the affected child (full trios) were genotyped. The analysis of the genotypes of the 251 trios using Transmission Disequilibrium Test (TDT) followed by False Discovery Rate (FDR) multi-test correction yielded 9 noteworthy genes, that are associated with SLE with FDR less than 0.5; two of these genes were highly significant, with FDR less than 0.05 [10].

Using Bayesian methodologies, the impact of pre-existing knowledge of a disease on the discovery of genes associated with the disease can be increased, as the posterior probability of association with the disease can be modified in accordance with its prior probability as reported by function2gene. The False Positive Report Probability (FPRP) measure is one such method which uses the prior probability of association, which can be calculated from the results of the keyword-based gene selection, to modify the posterior probability of association. Using Bayes' theorem $\left(P(A|B)=\frac{P(B|A)P(A)}{P(B)}\right)$, FPRP determines the probability of the null hypothesis (no association) being true given a test statistic greater than *Z*_α _(that is to say *p *≤ α), knowing power (1 - β), the prior probability of association (π), and the probability of the measured data given that the null hypothesis is true (*p*) [11]:

$$\text{FPRP}=P(H_{o}\text{ is true}|T>Z_{\alpha })=\frac{p(1-\pi )}{p(1-\pi )+(1-\beta )\pi }$$

One method of calculating prior probability based on the keyword based gene selection is to order the SNPs according to number of times they were returned by different keywords, taking into account the biological relevance of the SNP, and then apply a continuous function such that the higher ranked SNPs have a greater prior probability of association than the lower ranked SNPs, and the sum of the probability of association is the prior estimate of the total number of SNPs in the search believed to be associated with the disease. An alternative method is to order the SNPs in the same manner, and then place them in to different groups, assigning the same prior probabilities to each SNP in a group while controlling the sum of the prior probabilities assigned. For example, assuming 10,000 SNPs, 10 of which are believed to be associated, assign priors of π = 0.025 for the top 1%, 6.25 × 10^-3 ^to the next top 4%, 1.25 × 10^-3 ^to the next top 20%, and 3.33 × 10^-4 ^to the remaining 75%. In this manner the multiple testing effect is controlled while maximizing the effect of the prior available information. Applying FPRP [11] to the results of the TDT test with a prior assumption of 8 associated SNPs yielded 12 noteworthy genes, including all 9 obtained with the FDR corrections, and the same two significant genes [10].

An existing web-based program which is functionally similar to the methodology presented here is the Disease Candidate Gene search of SNPs3d. Using the keywords chosen by SNPs3d for three diseases, diabetes, pancreatic cancer, and Alzheimer disease, we have compared the results obtained by SNPs3d and Function2Gene in Table 2. The majority of high ranking genes returned by SNPs3d are also returned by Function2Gene, but Function2Gene returns a far greater number of genes.

**Table 2:** Comparison of Function2Gene and SNPs3d

	Returned by		Returned only by			
			
	Function2Gene	SNPs3d	Function2Gene	SNPs3d	Both	Total
Alzheimer's	2896	665	2399	168	497	3064
Diabetes	1744	881	1187	324	557	2068
Pancreatic Cancer	3283	755	2748	220	535	3503

Total	7923	2301	6334	712	1589	8645

Comparison of the number of genes returned by Function2Gene and SNPs3d for three diseases, showing the number of genes returned only by a Function2Gene or SNPs3d, and the number of genes returned by both search methodologies.

Future advancements of the approach presented here could be made by the use of more powerful literature mining techniques which would reduce (or even eliminate) the need for expert information on the nature, pathology, and biology of the disease to generate a list of keywords and discard spurious results. Such approaches would also reduce the reliance of this approach on the contents of stewarded fields in the databases, enabling novel associations as well as incorrect associations to be discerned. For example, Named Entity Recognition (NER) and Relationship Extraction (RE) could be used in tandem to elucidate connections between diseases and genes directly. NER identifies biologically-relevant entities (like genes and proteins) from literature using techniques such as hidden Markov models and dictionaries. Once entities have been identified, RE can identify the relationship and/or connection between entities using the proximity of entities (and the re-occurrence of entities in close proximity), along with rule base systems and full predicate/subject grammars [12-14]. It would then be possible to walk the relationship tree, using the probabilities between each node of the tree connecting specific genes and a disease (with intervening genes, proteins, and biological pathways in between), and then ordering the resultant genes by the probability of their connection which should be directly proportional to the prior probability of association.

## Conclusion

The use of available prior information to decrease the size of the problem space for gene identification in complex polygenic disorders is an as yet underutilized technique. The methodology and the programs presented here use data mining techniques to retrieve from a few databases a number of genes with high prior probability of being associated with the disease. The program also allows to order genes by relevance, thereby allowing the problem space to be greatly reduced, increasing the power of the study and thus increasing the likelihood of successfully finding associated genes.

## Availability and requirements

• Project name: Function2Gene

• Project homepage: 

• Operating system(s): Platform independent

• Programming Language: Perl

• License: GNU GPL v2 or later at your option

## Abbreviations

**FDR**: False Discovery Rate; **FPRP**: False Positive Report Probability; **NER**: Named Entity Recognition; **RE**: Relationship Extraction; **SLE**: Systemic Lupus Erythematosus; **SNP**: Single Nucleotide Polymorphism; **TDT**: Transmission Disequilibrium Test.

## Authors' contributions

DLA wrote the software described herein as well as the text of this article; COJ and RZ contributed to design of the software and the text of this article. All authors read and approved the final manuscript.
